# Assessing the Necessity of the Standardized Infection Ratio for Reporting Central Line-Associated Bloodstream Infections

**DOI:** 10.1371/journal.pone.0079554

**Published:** 2013-11-04

**Authors:** Daniel M. Saman, Kevin T. Kavanagh

**Affiliations:** 1 Essentia Institute of Rural Health, Division of Research, Duluth, Minnesota, United States of America; 2 Health Watch USA^sm^, Somerset, Kentucky, United States

## Abstract

This brief article presents results that support the contention that risk adjustment via the standardized infection ratio (SIR) for the reporting of central line-associated bloodstream infections (CLABSIs) may be no more predictive than standard rate adjustments utilizing CLABSIs per central line days (i.e., CLABSI rates). Recent data posted on the U.S. Department of Health and Human Services’ Hospital Compare website showed that nearly 70% of 1721 reporting hospitals with at least 1000 central line days had five or fewer infections during 2011. These hospitals had 39.3% of the total central line days and a significantly lower SIR than poorer performing hospitals with six or more CLABSIs (p<0.0001). In addition, 19 hospitals are presented which had central line days between 9000 to over 22,000 that also had zero to three CLABSIs. Some of these hospitals were university referral centers and inner city facilities. There was great variation of CLABSI cases among US hospitals. Evidence is mounting that all hospitals should be able to achieve a near zero incidence of CLABSIs and that these infections may in fact be near ‘never events’, which begs whether risk adjustment with the SIR is needed and whether it adds more information than does rate adjustment using CLABSI rates.

## Introduction

The standardized infection ratio (SIR) is a patient safety metric developed by the Centers for Disease Control and Prevention for comparing hospital outcomes across multiple locations by risk adjusting for differences in hospital size, teaching status, intensive care units (ICUs) size and type (which can be seen as a surrogate for patient severity) [1]. Currently, central line-associated bloodstream infections (CLABSIs) in hospital ICUs are major infections whose reporting is nationally mandated.  Data on hospitals’ observed CLABSI cases, central line days, predicted cases, and risk adjusted SIR are presented on the Hospital Compare website for consumer value-purchasing, with the SIR used for comparing hospitals to each other and to a “National Benchmark” [2].  

However, risk adjustment may be unnecessary for CLABSIs and may not add any more information than CLABSI rate adjustment using central line days, as many hospitals are reporting such few cases as to have us believe that CLABSIs should be considered near ‘never events’. Implementation of prevention protocols and checklists, as described by Berenholtz, et al.[3], can decrease the incidence of CLABSIs in hospital ICUs up to 66% [4,5]. These protocols, however, have not been uniformly adopted in all hospitals; a large number of hospital ICUs have achieved close to zero CLABSIs while in others it remains very high [6]. Our objective was to assess the necessity of risk adjustment using the SIR and compare the SIR to CLABSI rates by illustrating individual hospital examples and by analyzing hospitals grouped by the absolute number of CLABSI cases and comparing these groups’ total central line days and average SIRs. We hypothesized that the majority of hospital ICUs would have close to zero CLABSIs, and that risk adjustment using the SIR may not be necessary.

## Methods

This research was approved by the Essentia Health Scientific Review Board (SRB).  Hospital Compare is a nationwide public access database made available by the Centers for Medicare and Medicaid with infection counts and standardized infection rates by hospitals. Each reporting hospital consents its patients; thus, consent was not an issue for the authors as this study has over 1700 hospitals included. 

We created a histogram of CLABSIs by hospital frequency using Hospital Compare data for hospital ICUs with at least 1000 central line days to emphasize that CLABSIs should be close to zero regardless of risk factors or central line days (proxy for hospital size) (Figure) [2].  Out of 3617 hospitals in the Hospital Compare database, 1721 (47.6%) were included in the analysis; of those hospitals excluded from the analysis, 1120 hospitals had no data available because their predicted number of CLABSIs was less than one, 510 hospitals did not have an ICU location, and 266 hospitals had fewer than 1000 central line days. To be included in the NHSN dataset, a facility’s predicted (or expected) number of CLABSI—an estimate based on infections reported to NHSN during January 2006 to December 2008 [7]—had to be greater than or equal to one [8]. From this dataset we used three variables: Central line days, CLABSI observed cases, CLABSI predicted cases, and CLABSI SIR by hospital ICU.

A table was also created to show differences in the SIR between hospitals with a low number of CLABSIs and hospitals with higher CLABSI cases, as well as to compare the percent of CLABSI cases within each hospital group (e.g., 1-5 CLABSIs, 6-10 CLABSIs, etc.) to the percent of central line days within each hospital group. Two tail t-tests were performed comparing the different groups using Excel 2010 (Excel Microsoft, Redmond, CA 2010) on the SIR and the CLABSI rate using the 1-5 CLABSI group as the reference with significance set at p<0.05. Finally, we created a scatter plot and calculated an *R-square* value comparing the SIR to CLABSI rates. 

## Results

**Figure 1:**
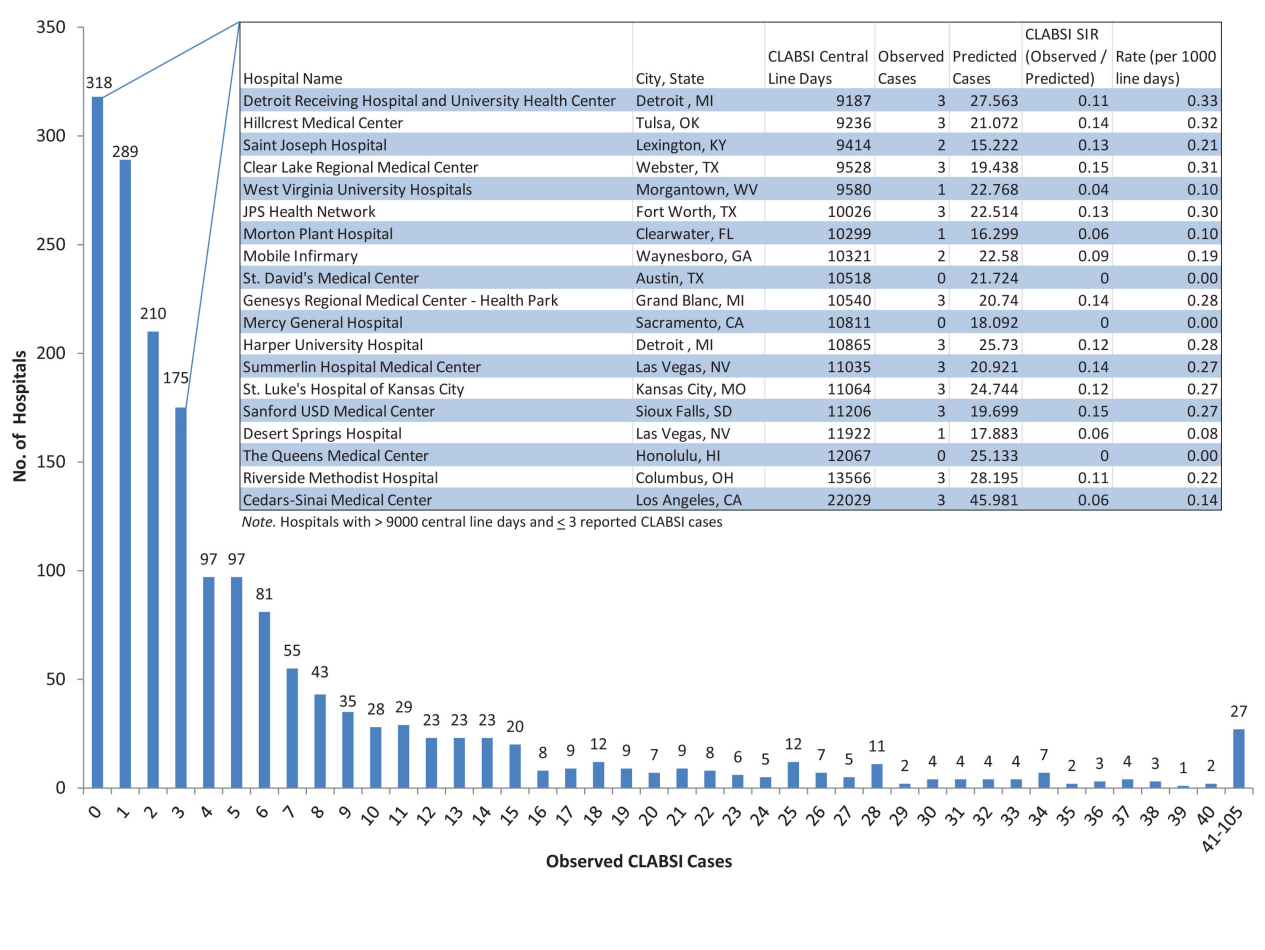
Histogram of observed CLABSI cases by hospital ICUs with at least 1000 reported central line days, 2011.

We found that among hospital ICUs with at least 1000 central line days, 18.5% (318/1721) reported zero CLABSI cases, and 68.9% (1186/1721) reported five or fewer CLABSIs during the collection period January 1, 2011, to December 31, 2011 (Figure 1).  The embedded table in Figure 1 is a listing of hospitals with greater than 9000 central line days and less than or equal to three CLABSIs.  Nineteen hospitals in the Hospital Compare database made the cut, including Cedars-Sinai in Los Angeles, CA, which reported 22,029 central line days and only three CLABSIs. The 19 hospitals represent 213,214 central line days, or 2.3% (213,214/9,328,140*100) of the total central line days, yet only 40 observed CLABSI cases, or 0.38% (40/10659*100) of the total CLABSI cases within the Hospital Compare database (n=1721). They also had a total of 436 predicted cases; a relatively large difference between the 40 observed cases. These hospitals demonstrate that facilities can indeed keep the number of patients who develop CLABSIs close to zero. 

The Table 1 shows descriptive statistics on hospitals grouped by number of CLABSIs (0, 1-5, 6-10, 11-15, 16-20, and 21-105). Among the 1721 hospitals with ICUs and at least 1000 central line days, there were a total of 10,659 observed CLABSIs, 9,328,140 central line days, an average of 5420 central line days, and a mean SIR of 0.56. There was also a mean CLABSI rate of 1.0 case per 1000 central line days and a total of 19,030 predicted cases. Hospitals with zero CLABSIs reported 7.9% of the total central line days. And hospitals with 1-5 CLABSis had 19.8% of the total number of observed CLABSI cases while disproportionately having 31.4% of the total number of central line days. The 21-105 CLABSIs hospital group had 41.4% of the total number of observed cases while disproportionately only having 26.8% of the total central line days. Moreover, the SIR and the CLABSI rate for the 1-5 CLABSIs group (SIR=0.56, CLABSI rate=0.93) was significantly lower than each of the other groups. As expected, the average number of central line days increased as the number of CLABSIs by hospitals increased.

**Table 1:** SIR, central line days, and CLABSI rate across hospital ICUs grouped by number of CLABSIs, 2011.

										Central line days				
	# Hospitals (%total)	Total Observed Cases (% total)	Total Predicted Cases (% total)	Mean SIR (SD)	Median SIR	Mean CLABSI Rate per 1000 line days (SD)	Median CLABSI Rate per 1000 line days	Total line days (% total)	Mean (SD)	Median	Q1 -25%	Q3 -75%	Low Range	High Range
0 CLABSIs	318 (18.5%)	0 (0%)	1234 (6.5%)	0 (0)	0	0 (0)	0	735196 (7.9%)	2312 (1487)	1899	1333	2786.25	1000	12067
1-5 CLABSIs	868 (50.4%)	2107 (19.8%)	5255 (27.6%)	0.56 (0.44) (ref)	0.44	0.93 (0.68) (ref)	0.74	2927803 (31.4%)	3373 (2247)	2681	1778	4400.25	1000	22029
6-10 CLABSIs	242 (14.1%)	1810 (17.0%)	2863 (15.0%)	0.93 (0.67) *	0.76	1.7 (1.1) *	1.44	1457736 (15.6%)	6024 (3367)	5351	3536	7466	1106	18573
11-15 CLABSIs	118 (6.9%)	1516 (14.2%)	2317 (12.2%)	0.94 (0.67) *	0.79	1.9 (1.1) *	1.56	1074475 (11.5%)	9106 (4661)	8392	5599	11158	1653	27939
16-20 CLABSIs	45 (2.6%)	808 (7.6%)	1429 (7.5%)	0.74 (0.43)**	0.69	1.6 (0.89) *	1.49	628557 (6.7%)	13968 (6744)	11856	8857	19374	3723	30675
21-105 CLABSIs	130 (7.6%)	4418 (41.4%)	5932 (31.2%)	0.87 (0.40)*	0.78	2.1 (0.95) *	1.77	2504373 (26.8%)	19264 (10681)	17842	11438	24125	5238	61359
Total	1721 (100%)	10659 (100%)	19030 (100%)	0.56 (0.55)	0.44	1.0 (0.98)	0.81	9328140 (100%)	5420 (6084)	3321	1923	6362	1000	61359

* p<0.0001

** p=0.0045

**Figure 2:**
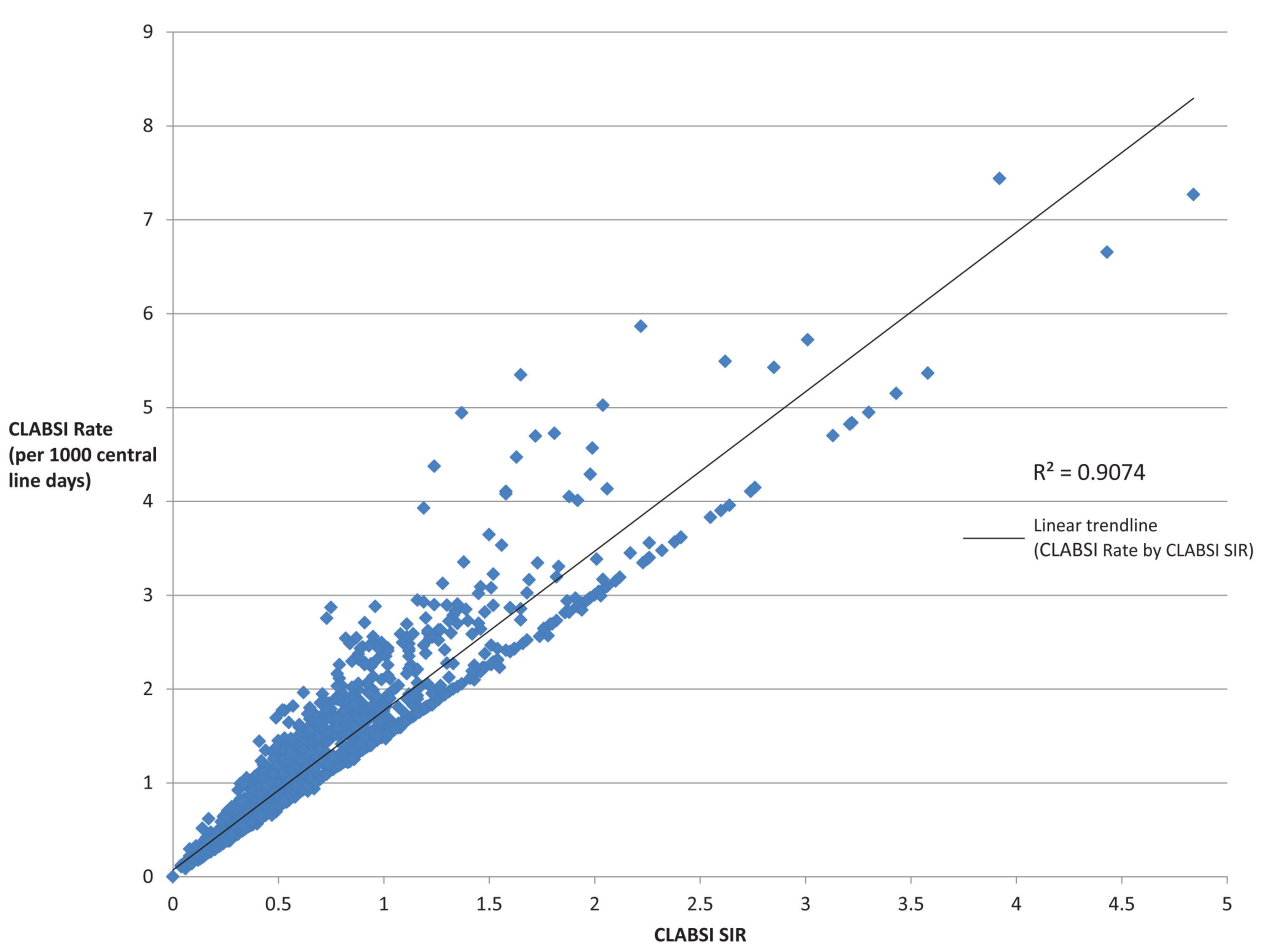
Scatter plot of CLABSI SIR by the CLABSI rate (per 1000 central line days) among hospital ICUs with at least 1000 reported central line days, 2011.

Figure 2 is a scatter plot of CLABSI rates (per 1000 central line days) by the CLABSI SIR among all hospitals in the dataset (n=1721). A linear trendline found an *R-square* value of 0.9074. 

## Discussion 

The national CLABSI SIR has shown a dramatic yearly decrease (2009, SIR=0.830; 2010, SIR=0.654; 2011, SIR=0.568) since its baseline collection period from 2006 to 2008 (SIR=1.0) [1,9,10].   However, effective prevention protocols have not been uniformly adopted by all facilities.  All the groups in the Table 1 had an SIR below 1.0 (i.e., all lower or no different from the national benchmark), and a majority of hospitals had five or fewer CLABSIs. Moreover, the average SIR among all the hospitals is lower than the National Benchmark. The National Benchmark was determined using old data and reflects old standards of performance, as evidenced by the mean SIR for all facilities of 0.56. A previous study by Saman et al., contends that the 2006-2008 national benchmark which facilities are compared to (e.g., better than, worse than, no different than) is out of date and does not reflect up-to-date infection control methods [6]. 

The vast majority (68.9%) of hospitals had below five CLABSIs. In many hospitals, 318 (18.5%), the number of CLABSIs was zero.  In addition, a third of the facilities in the Keystone Project in Michigan were able to achieve a level of zero [11]. Detractors of the belief that zero or near zero levels of CLABSIs are achievable in all facilities use two main arguments: The first is that facilities with a low number of CLABSI cases are those with a small number of central line days. The data in the Table 1 show that there is a definite increase in the number of CLABSIs as the number of line days increases. However, the rate of increase is less than the rate of increase in hospital central line days. In addition, as shown in Figure 1, a number of large facilities were able to achieve a level of zero CLABSIs. 

The second is that facilities with a small number of CLABSI are those that take care of patients with less acute disease. However, an analysis of the SIR—which adjusts for ICU type, a surrogate for patient severity and hospital level risk factors—in the Table 1 shows that facilities with five or less CLABSIs have a significantly lower SIR than those having six or more CLABSIs. This indicates that there are significant CLABSI risk factors other than patient severity of illness in facilities with a high number of CLABSIs. 

 As illustrated in the embedded table in Figure 1, many facilities report CLABSIs of zero or near zero, even in large major medical centers which have between 9,000 to 22,000 central line days and between 15 to 46 predicted cases. Future research should address several questions that are beyond the scope of this paper: Are the results from these high performing facilities, some of which are university medical centers which take care of the sickest patients, due to an improved safety culture and implementation of checklists and safety protocals? And with a culture of safety and dedicated adherence to safety protocols, will these low numbers of CLABSIs persist over time? If these facilities can maintain their total number of CLABSIs near zero, then it would be unlikely that chance plays a major role; and thus, low numbers of CLABSIs should be achievable by all facilities through adherence to safety protocols, limiting the value of risk adjustment of CLABSIs using the SIR. 

Figure 2 shows that hospital SIRs and CLABSI rates are highly correlated. This leads us to believe that CLABSI rates—even though they are not adjusted for facility type, bed size, and teaching institution—are just as good as SIRs and that the SIR adds little value to consumers beyond CLABSI rates alone. Further, given that most hospital ICUs are reporting five or fewer CLABSIs during 2011, overcorrecting for hospital characteristics using the SIR may only benefit those poorer performing institutions. 

Although there is not a definitive study from a large number of institutions, there have been reports emerging that once low numbers of CLABSIs are achieved, these levels are maintained. Nationally, the Table 1 shows that large facilities are capable of achieving a low number of CLABSIs. In addition, two case reports from Sutter Roseville Medical Center [12] and Bronson Methodist Hospital [13] illustrate that facilities can achieve and maintain low levels of CLABSIs for six years by implementing safety protocols. Moreover, Lipitz-Snyderman et al., found that within ICUs participating in the Michigan Keystone ICU Project, 60% maintained a median of zero CLABSIs for 12 or more months and 26% for 24 or more months [14], thus providing further reason to believe that achieving and maintaining zero CLABSIs is indeed possible. 

Evidence is mounting that risk adjustments are mostly useful for comparing poorly functioning hospitals and that sustained near zero infections are achievable even in facilities with high risk ICU patients (e.g., burn units) and those with a high number of central line days. Just as there is no risk adjustment for homicides in inner city hospitals or patient suicides—since the absolute numbers are so low and these events are viewed as largely avoidable events—we argue that CLABSI risk adjustment may not add more value than CLABSI rate adjustment using central line days. Risk adjustment may also give an impression that CLABSIs are primarily related to risk rather than the safety culture and protocols of a facility.    Because of the above, Consumer Reports does not risk adjust their highest rating and only awards it to those hospitals that have zero CLABSIs irrespective of hospital size, facility type, or the number of central line days [15]. 

### Limitations

 This study is limited by the type of data analyzed; mostly not validated. A previous study found that the reporting of CLABSI can be fraught with problems of bias; mainly that there is variation in the CLABSI surveillance definitions and in collecting data, and that this variation complicates the reported CLABSI SIR [16]. Another study noted a lack of agreement in classifying bloodstream infections by infection preventionists [17]. This could mean two serious data quality problems; 1) that the reported number of CLABSIs is artificially very low, and 2) that reported CLABSIs are often misclassified (bias in both directions). We agree there is a serious need for data validation and improved data quality in the collection and reporting of CLABSIs. However, in lieu of perfect data, we are compelled by our findings and maintain that the SIR is outdated, given that it is based on 2006-2008 data, before many facilities implemented additional safety protocols. In addition, the results of the Michigan Keystone ICU Project make it clear that hospitals can reduce and maintain their CLABSI rate, providing further evidence CLABSIs are indeed preventable. 

## Conclusion

Though this paper does not provide direct evidence against using the SIR correction, our objective was to legitimately question whether there is any added value from using the SIR correction over CLABSI rate adjustment using central line days. The prevention of CLABSIs is dependent upon the implementation of effective safety protocols and fostering a culture of safety within the institution. The necessity for risk adjustment of CLABSIs using the SIR needs to be reexamined.  Risk adjustments may be useful for non-rare events and to compare poorly performing facilities, but we contend that CLABSIs should be near ‘never events’ regardless of patient severity, hospital size, teaching status, ICU size and type. 
